# Use a Spoon as a Spade?: Changes in the Upper and Lower Alpha Bands in Evaluating Alternate Object Use

**DOI:** 10.3389/fpsyg.2018.01941

**Published:** 2018-10-23

**Authors:** Karolina Rataj, Deniece S. Nazareth, Frank van der Velde

**Affiliations:** ^1^Department of Psycholinguistic Studies, Faculty of English, Adam Mickiewicz University, Poznań, Poland; ^2^Department of Cognitive Psychology and Ergonomics, University of Twente, Enschede, Netherlands; ^3^Department of Psychology, Health, and Technology, Human Media Interaction, University of Twente, Enschede, Netherlands

**Keywords:** creativity, alternate use evaluation task, alpha, N400, sustained negativity, event-related desynchronization

## Abstract

Previous electrophysiological research on human creative cognition has related creative ideation to increased activity in the alpha band, an effect which mainly reflects increased general attentional demands. Research on alpha unrelated to creativity has revealed different functional roles of the upper (semantic processes) and lower (attentional processes) alpha sub-bands. At the same time, the need to dissect creative thinking into specific cognitive operations, such as, semantic processing, re-representation, or conceptual expansion has become evident. The main aim of the reported study was to test whether increased semantic processing demands linked to creating conceptual re-representations of objects required for evaluating alternate uses modulate activity in the upper and/or lower alpha sub-bands. For this purpose, we performed an alternate use evaluation task (AUeT), in which participants saw word pairs representing common uses, alternate uses, and unrelated word pairs, and evaluated whether a given use was common or uncommon (question 1), and how usable it was (question 2). Such an approach allowed us to examine the time-course of semantic processing involved in evaluating alternate uses. Additionally, the results could be contrasted with event-related potential (ERP) studies on creative language and semantic processing. We assumed that demands related to access and integration of semantic information needed to create a re-representation of objects (alternate uses) would be larger than in the case of common uses, which do not require creating a re-representation. This should be reflected in more activity in the alpha band in response to alternate than common uses, which was observed in the analysis of the upper alpha band over parieto-occipital sites. In the lower alpha band, more activity over the left than right anterior sites was observed for alternate uses, which might reflect increased attentional demands. Additionally, in the ERP analysis, alternate uses evoked larger N400 (400–500 ms) amplitudes than common uses, a pattern that extended to later time windows (500–1,000 ms). Overall, the results indicate increased semantic processing demands in alternate use evaluation, possibly linked to the creation of conceptual re-representations.

## 1. Introduction

Electrophysiological studies on creativity have often employed divergent thinking tasks, in which creative ideas are generated by participants in laboratory settings. In these tasks, participants are expected to be creative at will, and the recorded electrophysiological signal is compared between conditions on which they generate original and common ideas. Alpha-band oscillation analyses have frequently shown increases in the alpha-band activity on creative idea generation trials. Such results seem to indicate the involvement of general attentional mechanisms in creative idea generation. Recently, however, several authors have advocated dissecting creativity into more specific cognitive operations. Attention has been drawn to semantic processing and conceptual expansion (Kröger et al., 2013; Abraham, 2014), re-representation involving object replacement and object composition (Olteţeanu and Falomir, 2016), and to idea evaluation, which has been postulated to play an important role in creative ideation (Finke, 1995; Hao et al., 2016). Conceptual expansion in evaluation tasks has frequently been investigated in electrophysiological studies that have, however, rarely been explicitly considered in the discussion of electrophysiological markers of creativity. They have predominantly employed the event-related potential (ERP) method to examine meaning construction, e.g., in novel metaphor comprehension (Arzouan et al., 2007; Goldstein et al., 2012; Jankowiak et al., 2017; Rataj et al., 2018). Such studies have reported smaller N400 amplitudes evoked by literal utterances, whose comprehension involves meaning retrieval compared to novel metaphoric utterances, which require meaning construction. In the current study, we aimed to combine the two approaches to test whether changes in the alpha band and the N400 response index increased semantic processing demands in an alternate use evaluation task (AUeT). We assumed that semantic processing demands should be larger when participants evaluate creative object uses compared to common use evaluation. This stems from the need to create a re-representation of known objects and to establish novel semantic links to evaluate whether it is possible to use one object as another in a novel way. The AUeT we employed in the current study allowed us to examine both ERPs and changes in the alpha-band oscillations with fine temporal resolution needed to dissect alternate use evaluation into specific cognitive processes. Below, we will discuss the tasks and findings reported in previous electrophysiological studies on creativity that have reported the involvement of alpha in creative ideation. Next, we will briefly discuss the results of ERP studies on novel metaphor comprehension and their role in understanding creativity. Finally, we will present and justify the methodological modifications we decided to apply in the current study.

Divergent and convergent thinking tasks or conditions have been employed to investigate the relationship between alpha-band activity and creative cognition (Benedek et al., 2011; Jauk et al., 2012; Fink and Benedek, 2014). In divergent thinking tasks participants generate multiple solutions to a given problem, while in convergent thinking tasks, the number of generated solutions is limited, usually to one correct response (Guilford, 1967). Two divergent thinking tasks, the alternate uses task (AUT) and the word association task (WA), have most frequently been employed in electrophysiological research on creativity. In the AUT, participants are presented with a name or picture of an object and generate an alternate use of this object. In the WA task, participants are provided with a word and generate an uncommon/creative association with this word.

To compare the differences between creative and non-creative thinking, the two divergent thinking tasks have in some studies been contrasted with convergent thinking tasks, e.g., a mental arithmetic task, or tasks involving reciting the end word of common proverbs (Mölle et al., 1999; Razoumnikova, 2000; Krug et al., 2003; Shemyakina et al., 2007). However, such designs pose some difficulty in separating task demands from demands related to creative thinking. Additionally, the multitude of various tasks that have been employed makes it difficult to compare the results across various studies (Arden et al., 2010; Dietrich and Kanso, 2010; Jauk et al., 2012; Abraham, 2013). For this reason, recent studies have manipulated conditions within the same task rather than task types. For instance, Jauk et al. (2012) used the AUT and the WA task in the divergent and convergent modes. In the convergent mode, participants generated a common use of an object or word association, while in the divergent mode they generated an uncommon use of an object or an uncommon word association.

Importantly, instruction manipulations in divergent thinking tasks (Christensen et al., 1957; Harrington, 1975) have recently revived scholarly interest. So far used predominantly in behavioral research on divergent thinking and creativity, the manipulation involves instructing participants either to be creative, or to generate as many responses as possible. This manipulation evokes *a be-creative effect*, i.e., when participants are instructed to be creative (rather than be fluent), the originality of the generated uses increases, while the number of them decreases (Harrington, 1975; Chen et al., 2005; Nusbaum et al., 2014; Forthmann et al., 2016). In other words, instructions to be creative increase the novelty and reduce the fluency of generated responses. The electrophysiological correlates of this effect, however, remain to be examined.

The results of studies which have employed electroencephalography (EEG) to examine differences between divergent and convergent thinking have shown an event-related synchronization (ERS) in the alpha band in the divergent thinking modes. In the event-related synchronization (ERS, increase in power) and event-related desynchronization (ERD, decrease in power) analyses, the EEG power in a given frequency band in the reference interval, i.e., before the stimulus is displayed (pre-stimulus), is compared with the EEG power in this frequency band in the interval following stimulus onset (post-stimulus). In line with the recent views on alpha (Klimesch, 2012), increases in alpha power observed in divergent thinking modes can be interpreted as indexing top-down inhibitory control (Klimesch et al., 2007; Jensen and Mazaheri, 2010) and internally directed attention (Cooper et al., 2003; Benedek et al., 2011; Benedek, 2018).

Except for an increase in alpha power on divergent as compared to convergent conditions, differences in the topographical distribution of this effect have been reported in several studies. Little consistency, however, is present across these reports. While some studies have shown broadly distributed effects with no clear topographical differences between the creative and non-creative conditions (Mölle et al., 1999; Jaušovec, 2000), other studies have reported frontal alpha synchronization and linked it to high creative demands (Martindale and Mines, 1975; Fink and Neubauer, 2006). However, Benedek et al. (2011) demonstrated that frontal alpha synchronization might index general internal processing demands rather than creative thinking *per se*. In their study, the convergent thinking task involved finding an anagram solution to a target word, e.g., POST - STOP, and the divergent thinking task involved creating an original four word sentence based on the target string of letters (e.g., POST). For each sentence created by the participants, each letter of the target word constituted an initial letter of a word in the sentence. In this way, the critical word was the same in the creative and non-creative tasks, limiting between-task differences. Additionally, both tasks were used in a low internal processing demands condition, with the critical word presented on the screen for the duration of the trial, and in a high internal processing demands condition, with the critical word presented for 500 ms and then replaced with a mask. An event-related frontal alpha synchronization was found only in the high internal processing demands condition, however this result was not modulated by task type. This effect is in line with other studies that revealed a relationship between frontal alpha power and top-down attentional processes (Buschman and Miller, 2007), inhibitory top-down control (Lustenberger et al., 2015), and working memory demands (Sauseng et al., 2005). At the same time, Benedek et al. (2011) observed ERS in the alpha band over parietal regions only in the divergent thinking task under high internal processing demands, and interpreted it as indexing the recombination of semantically distant information needed to complete the divergent thinking task. This result points to the need for further research into the topographical patterns of alpha-band activity in research on creative cognition, with fine methodological manipulations of tasks and materials.

An important point of interest in EEG studies which examine the functional role of alpha is the distinction between lower and upper alpha sub-bands. In several previous studies, they have been associated with different mental operations, i.e., upper alpha has been linked to access and retrieval of semantic information, while lower alpha has been related to general attentional demands (Klimesch et al., 1997a,b, 2007; Klimesch, 1999; Doppelmayr et al., 2005; Freunberger et al., 2008). In EEG research on creativity, there seems to be little consistency in the results regarding the two alpha sub-bands. While some studies have shown increases in the upper alpha band (Fink et al., 2007, 2009; Shemyakina et al., 2007), other studies have revealed increases in the lower but not upper alpha band (Fink and Neubauer, 2006), or no differences in the observed patterns between the two sub-bands (Jaušovec, 2000; Benedek et al., 2011; Jauk et al., 2012). This lack of a clear distinction between the functional roles of the upper and lower alpha power in creativity might partially stem from the employed methodology. Namely, most EEG studies have examined alpha-band activity over time windows on the order of several seconds, during which participants generated creative responses. This approach has made the decomposition of creative thinking into specific cognitive processes a challenging task, as various stages of creative ideation remain impossible to separate (Abraham, 2014).

At the same time, cognitive processes play a crucial role in most recent models of creativity, which assume the involvement of at least two central processes in a creative act (Sowden et al., 2015). Such models, known also as the dual-process models of creativity, assume an interplay between the development of a novel idea and its evaluation (Basadur et al., 1982; Allen and Thomas, 2011). For example, according to one of such models, the Geneplore model (Finke, 1995), creative thinking involves (1) the generation of preinventive structures and (2) the evaluation of these structures. However, it remains difficult to identify these two stages in most EEG studies on creativity, partly due to long time windows used in the analyses. Although one ideal methodological solution to this conundrum is not easy to find, using both production and evaluation tasks might contribute to a better understanding of creative thinking by providing perspectives on different stages of creativity. The current study attempts to address this challenge by focusing on one of the stages proposed within the Geneplore model, i.e., creative idea evaluation.

The importance of examining idea evaluation has been emphasized in two recent studies. Hao et al. (2016) showed that evaluation of creative ideas is linked to activity in the upper alpha band, and that it can enhance creative thinking. In this study, participants generated original uses of everyday objects (AUT) in epoch 1. This epoch was followed by either a reflection (evaluation) task or a distraction task. In the reflection task, participants mentally evaluated the ideas they generated according to how original they thought these ideas were. As a control condition, a distraction task was used, in which participants provided typical characteristics of an object presented on the computer screen. Out of 20 AUT problems, 10 involved a reflection task, and 10 involved a distraction task, and a within-subject design was used. The reflection / distraction tasks were followed by epoch 2, in which participants worked on the same AUT problems as in epoch 1. This time they generated and reported the most original ideas that were not produced in epoch 1. The results revealed that when participants engaged in the evaluation task, they generated more original ideas than when they completed the distraction task. Most importantly, an enhancement in the upper alpha band over frontal sites was observed in the evaluation, but not the distraction condition. A similar enhancement was observed during idea generation after completion of the evaluation, but not the distraction task.

Another study that employed an evaluation task (the modified version of the alternate uses task) was an ERP study reported by Kröger et al. (2013). In this study, participants saw alternate uses, common uses, and unrelated word pairs, and evaluated them according to whether they thought a given use of an object was common or uncommon, and appropriate or inappropriate. The study reported differences between the three word pair categories in the N400 amplitudes. The N400 is a negative-going wave observed between 300 and 500 ms after critical word onset. First reports of the N400 effects revealed that the N400 amplitudes are larger for semantically anomalous than semantically congruent stimuli (Kutas and Hillyard, 1984). Later, semantic complexity was shown to modulate this effect, as novel meaningful stimuli evoked smaller N400 amplitudes when compared to novel meaningless stimuli (Chwilla et al., 2007). The N400 has been postulated to reflect the intersection between bottom-up processing of a stimulus and top-down activity in semantic memory. As a result of this intersection, an initial conceptual representation is created, which might be refined at later processing stages, if necessary (Kutas and Federmeier, 2011).

Subsequent reports revealed that the N400 amplitudes can be modulated by the degree of semantic difficulty and semantic novelty of the stimulus (Goldstein et al., 2012; Rataj et al., 2018). Kröger et al. (2013) found larger N400 amplitudes when participants evaluated creative than common uses of objects, which was interpreted as indicating the mismatch between world knowledge and the critical word. In the later time window (500–900 ms), sustained negativity amplitudes for alternate uses did not differ from those for common uses. This shift in differences was interpreted as reflecting a successful integration of both common and alternate uses. No topographical differences for these effects were reported. Alpha-band oscillations were not examined.

Importantly, investigating creative idea evaluation with the ERP technique places the electrophysiological examination of creativity in a broader context of a large number of ERP studies on creative language processing. One such instance is research on novel metaphor comprehension, in which participants perform a semantic judgment task, i.e., they evaluate whether novel metaphoric utterances, e.g., *a moldy theory*, and literal utterances, e.g., *a new theory* are meaningful or meaningless (Arzouan et al., 2007; Goldstein et al., 2012; Rataj et al., 2018). A frequently reported N400 effect, with novel metaphoric utterances evoking larger N400 amplitudes than literal utterances, calls for a direct comparison with research on creativity. This effect has been interpreted as indexing larger semantic processing demands linked to conceptual expansion needed to build the meaning of a novel metaphoric utterance, as compared to meaning retrieval in literal utterance processing. Conceptual expansion can be viewed here as an instance of re-representation that participants need to form to integrate the novel meaning with the previously established concept. Analogously, to evaluate a possible novel object use, participants need to build the re-representation, or broaden the conceptual representation of the two objects. This is obtained by retrieval and recombination of distant semantic information and creation of a novel link between the two remotely associated concepts.

An undeniable link between alpha oscillations and creative ideation that has been demonstrated in previous studies, the methodological challenges in EEG research on creative idea generation, together with the importance of idea evaluation in understanding creativity inspired us to design an EEG study, which examined alpha-band activity related to creative and noncreative content evaluation. Our main aim was to test whether changes in the upper and/or lower alpha sub-bands are modulated by the category of object use (common vs. creative). To this aim, we employed an alternate use evaluation task (AUeT), in which participants saw word pairs representing common and alternate uses of objects, as well as unrelated word pairs, and evaluated them by answering two questions: whether they thought a given use was common or uncommon (question 1), and whether it was usable, slightly usable, slightly unusable, or unusable to use one object as another (question 2).

Our main prediction was that evaluating alternate uses would be related to more activity in the alpha band than evaluating common uses. We based this prediction on results of previous studies which examined alpha ERS in creative ideation. Our study differs from previous reports in that we (1) employed an evaluation task and (2) performed the analyses in the time window between the critical word onset and 1,000 ms post-stimulus, over which time participants processed and integrated the meaning of the presented uses. Several previous studies on semantic memory have employed such analyses and showed an overall ERD in the alpha band peaking around 500 ms, which was interpreted as reflecting active stimulus processing (Klimesch et al., 1997a,b; Bastiaansen et al., 2002, 2008). An important pattern has been observed in some of these studies, i.e., the more integrated the semantic information was, the larger alpha ERD was observed. Thus, for information that was not well semantically integrated, reduced ERD in the alpha power band, indicating more alpha-band activity, was reported (Klimesch et al., 1997a, 2007; Klimesch, 1999, 2011, 2012). Hence, we expected that alternate uses in our study (representing less integrated information) would be related to such a reduction in ERD when compared to common uses. We aimed to examine these differences in both the upper and lower alpha sub-bands, as differences between them have been reported in several previous studies (Shemyakina et al., 2007; Fink et al., 2011; Fink and Benedek, 2014; Hao et al., 2016). In the ERP analysis, we expected to replicate the N400 effect observed in the study by Kröger et al. (2013) with alternate uses evoking larger N400 amplitudes than common uses. In the later time window (between 500 and 1,000 ms), we predicted that sustained negativity would be observed reflecting increased working memory load due to (1) a delayed response procedure, which required participants to maintain the information in working memory before the evaluations could be performed and (2) task complexity, as participants responded to two questions.

## 2. Methods

### 2.1. Participants

Twenty-three students of psychology at the University of Twente, the Netherlands participated in the experiment for course credits or monetary compensation. Data from one participant were excluded from further analysis due to excessive blinking. Among the 22 participants, 13 were female (*M*_*age*_ = 21, range 18–28). Twenty one participants were right-handed, with mean laterality index of 87 on a scale ranging from 100 (right handed) to –100 (left handed), and one participant was close to the middle of the scale, with the laterality index of –33, as measured by the Handedness Questionnaire adapted from Oldfield (1971) and modified by Mark Cohen in 2008 (available at: http://www.brainmapping.org/shared/Edinburgh.php). All participants were native speakers of Dutch, had normal or corrected to normal vision, and no history of neurological disorders. Each participant signed an informed consent form prior to participation.

### 2.2. Materials

Three experimental conditions used in the current study were word pairs representing common uses, alternate uses, and unrelated word pairs. A set of 56 word pairs was created by the experimenters for each experimental condition. The critical word was the same across all three conditions. The critical words were Dutch nouns, with the mean log10 word frequency of 2.93 (range: 2.65–3.47) in SUBTLEX-NL, a database of Dutch word frequencies (Keuleers et al., 2010). The mean number of characters of the critical words was 6.88 (range: 5–12). To ensure that the experimental word pairs met the criteria of the three categories, a web-based normative study was conducted prior to the EEG experiment. Based on its results, 52 word pairs per condition were included in the final set of experimental materials. Due to the need to construct a large number of word pairs representing the uses (156 in total), out of the 208 words used in the final set, 13 words (6%) were names of buildings (e.g., shelter, castle, hospital) and places (e.g., golfcourse or park). Eighty-two native speakers of Dutch (55 female, *M*_*age*_ = 26, range 18–67) not involved in the EEG experiment participated in the normative study. Three versions were constructed and the word pairs were counterbalanced across the three versions of the survey so that no critical word was repeated within a given version. Each participant completed one version and evaluated two blocks. In Block A, participants rated how common they thought it was to use one object as another on a 7-point rating scale, where 1 represented very common and 7 very uncommon uses. Only common and alternate uses were tested in this block. In Block B, participants rated how usable they thought it was to use one object as another on a 7-point rating scale, where 1 represented very usable and 7 very unusable. All three conditions were rated in this block. The order of blocks within a version was counterbalanced. Of the 82 participants, 27 completed version 1, 28 version 2, and 27 version 3. Data from four participants were excluded from the analysis as their means were more than three standard deviations away from the group mean.

In the analysis of the scores obtained on the common uses scale, one item from the set of alternate uses was excluded due to too low scores on the scale. Participants evaluated this use as common rather than uncommon, contrary to experimenters expectations. The analysis of the remaining word pairs revealed that the word pairs defined as common uses by the experimenters were evaluated as more common (*M* = 3.58, *SE* = 0.06) than the alternate uses (*M* = 5.82, *SE* = 0.07), (*p* < 0.001, *d* = 2.98).^1^ It is important to note that the common uses examined here were not rated as very common, as our intention was to avoid using word pairs in which one word in the pair would evoke strong expectations about the following word. This was crucial, as the N400 component has been shown to be sensitive to anticipation effects. For this reason, the common uses examined in the reported experiment are more common than the alternate uses, but not very common in a general context.

The analysis of the scores obtained on the usability scale revealed that three items from the set of alternate uses received too low or high scores. Two common uses were evaluated by participants as unusable, and two unrelated word pairs were evaluated as usable. These word pairs were excluded from the set and the analysis of the remaining word pairs revealed that common uses were evaluated as more usable (*M* = 2.41, *SE* = 0.07) than the unrelated word pairs (*M* = 6.13, *SE* = 0.05), (*p* < 0.001, *d* = 5.86). Moreover, a graded effect was observed with the alternate uses (*M* = 4.02, *SE* = 0.10) falling in between the common uses (*p* < 0.001, *d* = 3.01) and unrelated word pairs (*p* < 0.001, *d* = 2.79). Based on the results of the normative study, 52 alternate uses (CR), 52 common uses (CM) and 52 unrelated word pairs (UN) were selected for the final set of experimental stimuli (see Table 1 for examples).

**Table 1:** Examples of common uses, alternate uses, and unrelated word pairs in Dutch. Translations to English are provided in italics. Each critical word is preceded by a word representing a common use, an alternate use, and an unrelated word.

**Common use**	**Alternate use**	**Unrelated word pair**	**Critical word**
bloemen	vogelnest	viool	kroon
*flowers*	*nest*	*violin*	*crown*
aftershave	mosterd	trompet	parfum
*aftershave*	*mustard*	*trumpet*	*perfume*
t-shirt	gordijn	emmer	handdoek
*t-shirt*	*curtain*	*bucket*	*towel*
glas	tanden	kaas	diamanten
*glass*	*teeth*	*cheese*	*diamonds*

### 2.3. Procedure

The procedure of the experiment followed the guidelines for research with human participants and was approved by the Ethics Committee at the Faculty of Behavioral Sciences at the University of Twente, the Netherlands. Prior to the experiment, all participants signed informed consent. The experiment was carried out in the Laboratory of Behavioral Sciences at the University of Twente. The EEG signal was recorded from 64 active electrodes using an actiCAP system (Brain Products GmbH, Gilching, Germany) and Brain Vision Recorder software.

Once the EEG application procedure had been completed, the participant sat in front of the computer screen in a darkened and quiet room. Participants completed the alternate use evaluation task (AUeT), in which they evaluated pairs of words according to how common and usable the particular use of an object was according to them. To this aim, two questions were presented after the presentation of a given word pair.

**Figure 1:**
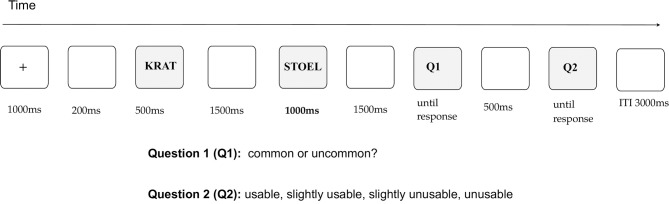
The sequence of stimuli together with exposure times are displayed. Participants answered two questions: whether a given use appeared common or uncommon to them (question 1), and how usable they thought a given use was (question 2).

Each participant first saw a fixation cross that remained on the screen for 1,000 ms and was followed by a blank screen (200 ms). Next, the first word of the word pair, e.g., *a curtain*, was presented for 500 ms, followed by a blank screen (1,500 ms), and the second (critical) word of the word pair, e.g., *a towel* (1,000 ms). In relation to the classical AUT, where participants generate original uses of objects, the first word of each word pair in the current study, e.g., *a curtain*, was the name of the object for which participants would need to find a novel use in the AUT. The critical word in our design was the use of the object, e.g., *a towel*, here however this use had been generated participants evaluated it. For this purpose, when the critical word disappeared, a blank screen was presented for 1,500 ms, after which Question 1 (Q1) was displayed. To answer this question, participants evaluated whether it was common or uncommon to use one object (word 1), e.g., *a curtain*, as another (word 2 / critical word), e.g., *a towel*. The question remained on the screen until the participants responded. Finally, a blank screen was presented for 500 ms, preceding the second question (Q2), which remained on the screen until participants' response. To answer this question participants decided whether the presented use was very usable, slightly usable, slightly unusable, or very unusable. Participants were explicitly informed that except for word pairs to which they could respond by indicating common (Q1)— slightly / very usable (Q2) and those to which they could respond uncommon (Q1) — slightly/very unusable (Q2), the option uncommon (Q1) — slightly / very usable (Q2) was also possible. The inter-trial interval was a blank screen displayed for 3,000 ms (see Figure 1). ERPs were time-locked to the onset of the critical word.

156 word pairs were presented to each participant. The word pairs were counterbalanced in three blocks, so that no critical word was repeated within a given block. Also, the order of blocks and the order of word pairs presented in each block was randomized across participants. Each participant was randomly assigned to a given order of blocks and completed a practice block prior to the three experimental blocks.

### 2.4. EEG recording and analysis

EEG signals were recorded from 64 active Ag/AgCl electrodes (Brain Products) located at the standard extended 10/20 positions with the ground placed at AFz. For blink artifacts, the electrooculography was recorded bipolarly from above and below the right eye (vEOG). For horizontal eye movements bipolar electrooculography was used, situated horizontally from positions next to the outer rims of the eyes. EEG data were amplified by a QuickAmp amplifier (BrainProducts GmbH) with average reference and filtered with a lowpass filter (cutoff frequency = 140 Hz). Signals were sampled at 500 Hz per channel and electrode impedance was maintained below 5 kΩ for all electrodes. All signals were stored on the computer for offline analyses.

Offline data analyses were conducted using Brain Vision Analyzer software (Version 2.1., Brain Products GmbH, Munich, Germany). For the analyses of event-related potentials, the raw EEG data were filtered with a Butterworth Zero Phase Filter with a low cutoff of 0.5 Hz and a high cutoff of 30 Hz. Next, data were segmented from 100 ms before stimulus onset to 1,000 ms afterward. A baseline correction of -100 to 0 ms was applied. Finally, trials with artifacts were removed (gradient criterion: 50 μV/ms, Max-Min criterion: +/- 150 μV, low activity criterion: 0.1 μV per 50 ms). Ocular artifacts were corrected by Gratton & Coles method (Gratton et al., 1983) as implemented in Brain Vision Analyzer 2.1.

25 electrodes were selected for the statistical analysis with three factors: the anterior-posterior axis, laterality (the left-midline-right orientation), and word pair type (common uses vs. alternate uses vs. unrelated word pairs). For the anterior-posterior axis the following electrodes were selected for each level: frontal (F3, F1, Fz, F2, F4), fronto-central (FC3, FC1, FCz, FC2, FC4), central (C3, C1, Cz, C2, C4), centro-parietal (CP3, CP1, CPz, CP2, CP4), and parietal (P3, P1, Pz, P2, P4) electrodes. The laterality factor included: F3, F1, FC3, FC1, C3, C1, CP3, CP1, P3 and P1 (left), Fz, FCz, Cz, CPz, Pz (midline), F4, F2, FC4, FC2, C4, C2, CP4, CP2, P4, and P2 (right).

In the acquired dataset, the following ERP components were identified based on visual inspection of the data: the P1 and N1, exogenous perceptual components, were observed over the posterior sites. Next, negativity was observed around 300 ms after critical word onset, peaking at around 400 ms after stimulus onset. The waveforms for the three conditions diverged after 400 ms after critical word onset. Based on this observation as well as previous research on semantic processing that showed differences in the early (around 300–400 ms) and late (around 400–500 ms) N400 time windows (see e.g., Lai et al., 2009), we performed the analysis of the N400 in these two time windows. This negativity continued until 1,000 ms and was most clearly visible over the frontal and fronto-central sites. The analysis was, thus, performed in three time windows: (1) between 300 - 400 ms (the early N400 time window), (2) between 400 - 500 ms (the late N400 time window), and between 500 and 1,000 ms (sustained negativity). To avoid possible bias stemming from brain activity associated with correct responses rather than stimulus category (VanRullen, 2011), we performed the statistical analyses on all responses. Additionally, this approach offers good signal to noise ratio, as the number of trials across all three conditions is the same.

Anterior-posterior × laterality × word pair type analyses of variance were performed in all three time windows. Based on the study reported by Kröger et al. (2013), we expected that in all time windows the largest negativity will be observed for unrelated word pairs, followed by alternate uses, which in turn would be followed by common uses. For this reason, for all main effects and interactions that were significant, we performed planned comparisons. Since three comparisons were performed in each case, we corrected for multiple comparisons by setting the significance level at α = 0.02.

For the time-frequency analysis, the raw EEG signal was filtered with the Butterworth Zero Phase Filter with a low cutoff of 0.5 Hz and a high cutoff of 30 Hz. Data were segmented from 1,000 ms before critical word onset to 1,000 ms afterward. A baseline correction of –100 to 0 ms was applied. Trials with artifacts were removed (gradient criterion: 50 μV/ms, Max-Min criterion: ± 150 μV, low activity criterion: 0.1 μV per 50 ms). Ocular artifacts were corrected by Gratton & Coles method (Gratton et al., 1983) as implemented in Brain Vision Analyzer 2.1.

Two types of analysis were performed. First, we performed the wavelet analysis on single trials. In this analysis we examined total power (both induced and evoked). A complex Morlet with Gabor normalization was applied (*c* = 5) on single trials for each participant. We extracted the power (in μV^2^) for seven frequency bands: lower theta (2.40–3.60 Hz), mid theta (3.30–4.93 Hz), upper theta (4.52–6.78 Hz), lower alpha (6.20–9.30 Hz), upper alpha (8.50–12.75 Hz), lower beta (11.66–17.49 Hz), and upper beta (16.00–24.00 Hz). Next, the trials were averaged per participant and condition. Second, the time-frequency analysis was performed on the ERPs, so trials were first averaged per participant and condition, and then the wavelet analysis was performed on these averages for each of the seven frequency bands. In this way, we calculated the evoked power.

To calculate the event-related desynchronization (ERD), we used the formula proposed by Pfurtscheller and Da Silva (1999), in which the percentage change in a given power band is the difference in power between the post-stimulus interval and pre-stimulus (reference) interval divided by the power in the reference interval, and multiplied by 100 (i.e., ((post-stimulus interval − pre-stimulus interval)/pre-stimulus interval) × 100). In the current study, the reference interval was selected between 300 ms before stimulus onset until 0 ms (stimulus onset), and the post-stimulus interval was selected between 400 and 1,000 ms after stimulus onset.

Anterior-posterior × laterality × word pair type analyses of variance were performed on the obtained ERD values. Separate analyses for the total power (induced and evoked) and evoked power were carried out for the upper and lower alpha bands. Supplementary analyses for the five remaining frequency bands (lower theta, mid theta, upper theta, lower beta, and upper beta) were performed to provide reference for previous and future work on the relationship between frequency bands other than alpha and creativity. Clear links between specific cognitive processes involved in creativity and the different frequency bands have not yet been well established, for which reason we find it important to report the complete results here.

For all analyses, Greenhouse–Geisser correction was applied, when necessary. Effect size is reported using partial eta squared ($\eta_{p}^{2}$), eta squared (for one-way ANOVA (η2)), and Cohen's *d*. The partial eta squared values should be interpreted in line with the following guidelines: 0.01 = small effect, 0.06 = a moderate effect, and 0.14 = a large effect (Cohen, 1988). For Cohen's *d*, the suggested interpretation is: 0.2 = small effect, 0.5 = a moderate effect, and 0.8 = a large effect (Cohen, 1988).

## 3. Results

### 3.1. Behavioral results

Since the distribution of reaction times was not normal, natural logarithms were computed for the obtained average reaction time values per participant and condition, and the statistical analyses were performed on these transformed values for both questions. Two analyses of the reaction time data were performed. First, a one-way ANOVA with word pair type as a factor (common uses vs. alternate uses vs. unrelated word pairs) was conducted on the categories identified by the EEG study participants. The second ANOVA was performed on the correct responses of the EEG participants for the categories established in the normative studies. For Question 1, the analysis of the three stimulus categories according to EEG study participants' choice revealed a main effect of word pair type [*F*_(2, 42)_ = 20.76, *p* < 0.001, η^2^ = 0.50]. Bonferroni corrected pairwise comparisons showed that reaction times recorded for common uses (*M* = 1,256, *SE* = 87) were longer than those recorded for alternate uses (*M* = 1,059, *SE* = 92) (*p* < 0.05, *d* = 0.62) and unrelated word pairs (*M* = 889, *SE* = 70) [*p* < 0.001, *d* = 1.29]. Alternate uses evoked longer reaction times than unrelated word pairs (*p* = 0.001, *d* = 0.92).

The analysis of correct responses to Question 1 yielded a similar main effect of word pair type [*F*_(2, 42)_ = 15.73, *p* < 0.001, η^2^ = 0.43]. Bonferroni corrected pairwise comparisons showed that reaction times to common uses (*M* = 1,205, *SE* = 83) were longer than those to alternate uses (*M* = 1,007, *SE* = 117) (*p* = 0.02, *d* = 0.63), which however differed only marginally from those for unrelated word pairs (*M* = 830, *SE* = 65) (*p* = 0.07, *d* = 0.52). There was a significant difference between the reaction times recorded for common uses and unrelated word pairs (*p* < 0.001, *d* = 1.23). The analyses of the responses to Question 2, for both the responses based on participant determined categories and the correct responses did not yield any significant effects (*p* > 0.05).

A one-way ANOVA with word pair type as a factor was also performed on response expectancy, in which we checked whether the participants of the EEG experiment agreed in their evaluations with the normative data study participants. In line with our predictions, a main effect of word pair type was found [*F*_(2, 42)_ = 59.60, *p* < 0.001, η^2^ = .74], with 91% of expected responses recorded in response to unrelated word pairs, which differed significantly from common 44% (*p* < 0.001, *d* = 2.88) and alternate uses 49% (*p* < 0.001, *d* = 2.05). Common and alternate uses did not differ (*p* > 0.05). Together with the reaction time data, the observed effects indicate that response difficulty was larger for common and alternate uses than for the unrelated word pairs.

### 3.2. EEG results: event-related potentials

#### 3.2.1. N400

##### 3.2.1.1. Early time window (300–400 ms)

In the early N400 time window, no main effects or interactions were observed [*p* > 0.05].

##### 3.2.1.2. Late time window (400–500 ms)

**Figure 2:**
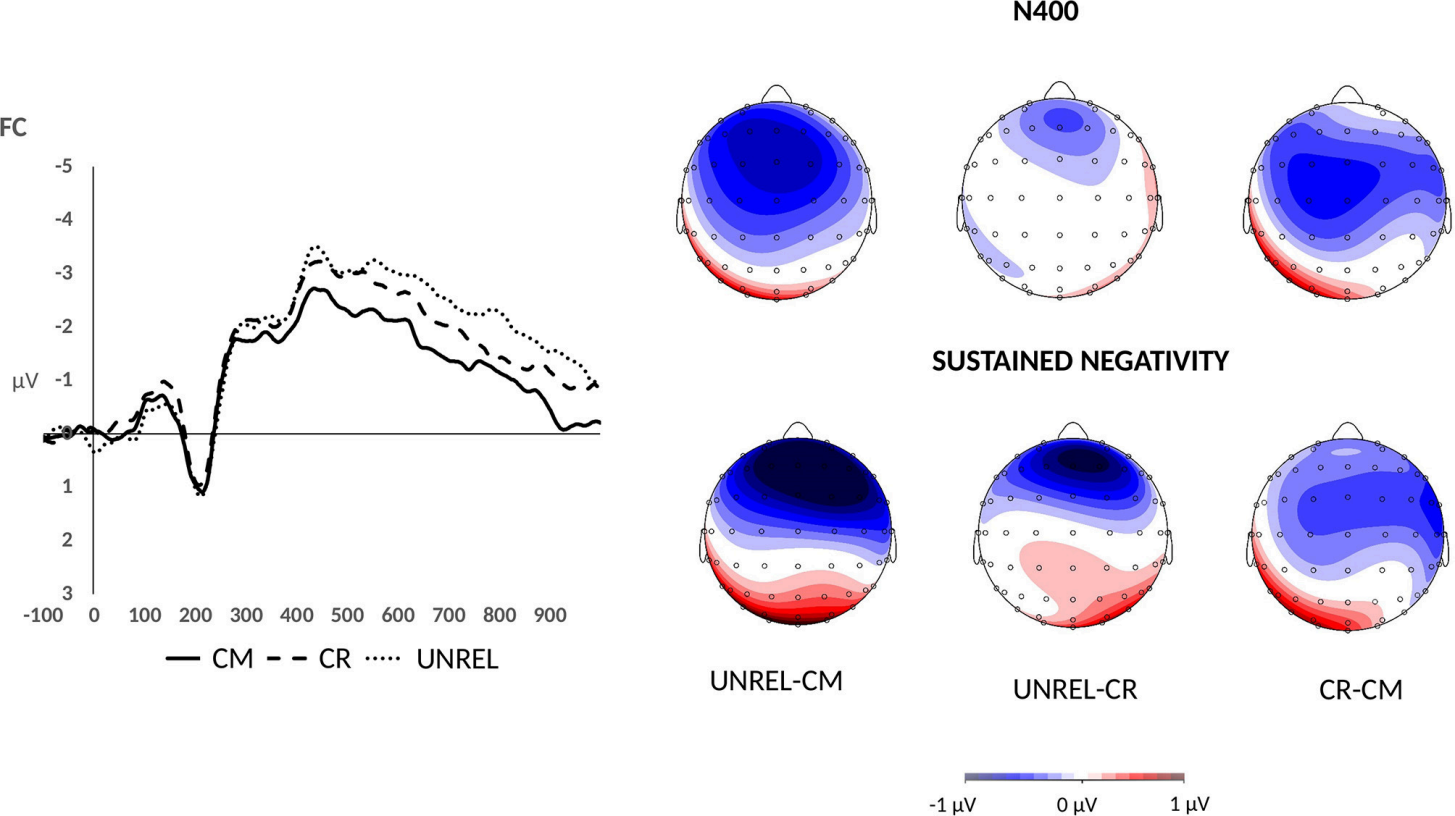
Grand average ERPs for common uses (solid line), creative (alternate) uses (dashed line), and unrelated word pairs (dotted line) over the fronto-central electrodes. Difference wave maps illustrate the N400 effect (400–500 ms) and the sustained negativity effect (500–1,000 ms). The maps were derived by subtracting the common use condition (CM) from the unrelated condition (UNREL), the alternate use condition (CR) from the unrelated condition (UNREL), and the common use condition (CM) from the alternate use condition (CR).

In the late time window, a main effect of word pair type was observed [*F*_(2, 42)_ = 3.81, *p* < 0.05, $\eta_{p}^{2}$ = 0.15]. One-tailed *t*-tests showed that the mean amplitudes for unrelated word pairs (*M* = −2.07, *SE* = 0.26) were significantly more negative than those for common uses (*M* = −1.68, *SE* = 0.26) (*p* < 0.01, *d* = 0.58). Furthermore, the difference between mean amplitudes for alternate uses (*M* = −1.96, *SE* = 0.25) and common uses was marginally significant (*p* = 0.03, *d* = 0.40). Finally, the difference in mean amplitudes for unrelated and alternate uses did not reach significance (*p* > 0.05). At the same time, a linear effect was found with unrelated word pairs evoking the largest N400 amplitudes, followed by alternate uses, which were followed by common uses [*F*_(1, 21)_ = 7.5, *p* = 0.01, $\eta_{p}^{2}$ = 0.26]. The difference wave maps and mean amplitude plots are presented in Figure 2. No interactions with word pair type were observed.

#### 3.2.2. Sustained negativity (500–1,000 ms)

Between 500 and 1,000 ms after stimulus onset, a main effect of word pair type was marginally significant [*F*_(2, 42)_ = 3.05, *p* = 0.06, $\eta_{p}^{2}$ = 0.13]. Moreover, an anterior-posterior axis × word pair type interaction was observed [*F*_(8, 168)_ = 5.12, *p* = 0.005, $\eta_{p}^{2}$ = 0.20]. To deconstruct this interaction, separate analyses were performed on the data recorded over frontal, fronto-central, central, centro-parietal, and parietal electrodes. Analyses over central, centro-parietal, and parietal electrodes did not reveal a main effect of word pair type (*ps* > 0.05). Maximal amplitudes were observed over the fronto-central sites, where a main effect of word pair type was present [*F*_(2, 42)_ = 7.06, *p* < 0.01, $\eta_{p}^{2}$ = 0.25]. The pattern resembled the one observed in the late N400 time window. One-tailed *t*-tests on mean amplitudes recorded over the fronto-central sites revealed that unrelated word pairs evoked more negative amplitudes (*M* = −2.35, *SE* = 0.36) than common uses (*M* = −1.52, *SE* = 0.32) (*p* < 0.01, *d* = 0.68). Alternate uses evoked intermediate amplitudes (*M* = −2.01, *SE* = 0.34), and differed from common uses [*p* = 0.02, *d* = 0.49]. The difference between mean amplitudes for unrelated word pairs and alternate uses was marginally significant [*p* = 0.04, *d* = 0.38]. A significant linear effect was found [*F*_(1, 21)_ = 10.17, *p* < 0.01, $\eta_{p}^{2}$ = 0.33]. A similar pattern was found over the frontal sites with a main effect of word pair type [*F*_(2, 42)_ = 9.78, *p* < 0.001, $\eta_{p}^{2}$ = 0.32], and one-tailed *t*-tests showing larger amplitudes for unrelated word pairs (*M* = −1.99, *SE* = 0.40) than the common uses (*M* = −0.90, *SE* = 0.34) (*p* = 0.001, *d* = 0.75) and alternate uses (*M* = −1.20, *SE* = 0.35) [*p* = 0.001, *d* = 0.78]. Although common and alternate uses did not differ (*p* = 0.09), a significant linear effect [*F*_(1, 21)_ = 12.23, *p* < 0.01, $\eta_{p}^{2}$ = 0.37] was found. The difference wave maps are presented in Figure 2.

#### 3.3. EEG results: event-related desynchroniztion

To check whether any differences in the alpha ERD were observed between the three experimental conditions, we performed the time-frequency analysis and examined the time window between 400 and 1,000 ms, within which the graded sustained negativity effect was found. Both upper and lower alpha bands were examined. The analysis was performed on single trials that were later averaged (evoked and induced power) and on the ERPs (evoked power).

**Figure 3:**
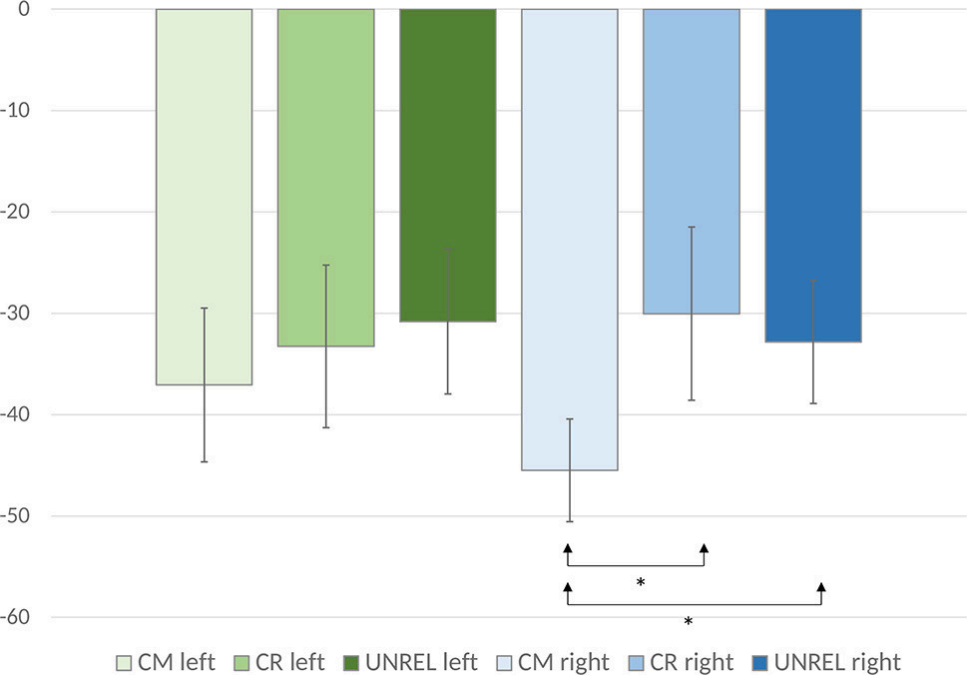
Mean upper alpha ERD values are displayed for the common, creative (alternate), and unrelated conditions. Error bars represent standard error values. The mean ERD values for the left electrodes were calculated by averaging the ERD values for PO3 and PO7. The mean ERD values for the right electrodes were calculated by averaging the ERD values for PO4 and PO8.

The analysis of evoked power did not yield significant results in the upper or lower alpha bands. In the analysis of the induced and evoked power, an anterior-posterior × laterality × word pair type ANOVA showed a marginally significant main effect of word pair type [*F*_(2, 42)_ = 2.91, *p* = 0.07, $\eta_{p}^{2}$ = 0.12], and a laterality × word pair type interaction [*F*_(8, 168)_ = 2.54, *p* = 0.05, $\eta_{p}^{2}$ = 0.11]. Also, an anterior-posterior × laterality × word pair type interaction [*F*_(40, 840)_ = 1.43, *p* < 0.05, $\eta_{p}^{2}$ = 0.06] was observed. To deconstruct this interaction, we performed separate analyses for each of the anterior-posterior levels. Analyses of the data recorded over frontal, fronto-central, central, and centro-parietal sites did not reveal significant main effects or interactions, (*ps* > 0.05). Only the analysis of data recorded over parieto-occipital sites showed a main effect of word pair type [*F*_(2, 42)_ = 5.02, *p* = 0.01,$\eta_{p}^{2}$ = 0.19] with common uses evoking a significantly larger ERD (*M* = −38.49, *SE* = 6.35) than alternate uses (*M* = −27.89, *SE* = 8.33) [*p* < 0.05, *d* = 0.63] and unrelated word pairs (*M* = −29.46, *SE* = 6.55) (*p* = 0.05, *d* = 0.55). The difference between alternate uses and unrelated word pairs did not reach significance. Analyses of the data recorded over parietal sites revealed a marginally significant main effect of word pair type [*F*_(2, 42)_ = 2.94, *p* = 0.06, $\eta_{p}^{2}$ = 0.12]. Although the ERD pattern was similar as in the analysis of the data recorded over parieto-occipital sites (common uses: *M* = −22.83, *SE* = 5.41, alternate uses: *M* = −12.88, *SE* = 6.18, unrelated word pairs: *M* = −17.24, *SE* = 6.02), pairwise comparisons did not show significant differences. To inspect possible laterality effects over the parieto-occipital sites, where the effect was most pronounced, the analyses of individual electrodes were conducted. They showed no main effects over the left and midline sites (PO7, PO3, and POz, [*ps* > 0.05]), while main effects were observed over the right electrode sites (PO4 [*F*_(2, 42)_ = 6.30, *p* < 0.01, η^2^ = 0.23], PO8 [*F*_(2, 42)_ = 6.76, *p* < 0.01, η^2^ = 0.24]) (see Figure 3). To test between-condition differences, we computed mean values for the data recorded over these two electrodes for each condition and performed a one-way ANOVA. The results showed a main effect of word pair type [*F*_(2, 42)_ = 7.08, *p* < 0.01, η^2^ = 0.25], with common uses evoking the largest upper alpha ERD (*M* = −45.50, *SE* = 5.07), which differed significantly from alternate uses (*M* = −30.04, *SE* = 8.55) (*p* = 0.02, *d* = 0.65) and unrelated word pairs (*M* = −32.84, *SE* = 6.07) (*p* = 0.001, *d* = 0.93). Alternate uses did not differ from unrelated word pairs (*p* > 0.05), but a significant linear effect was observed (*F*_(1, 21)_ = 18.91, *p* < 0.001, η^2^ = 0.47].^2^

**Figure 4:**
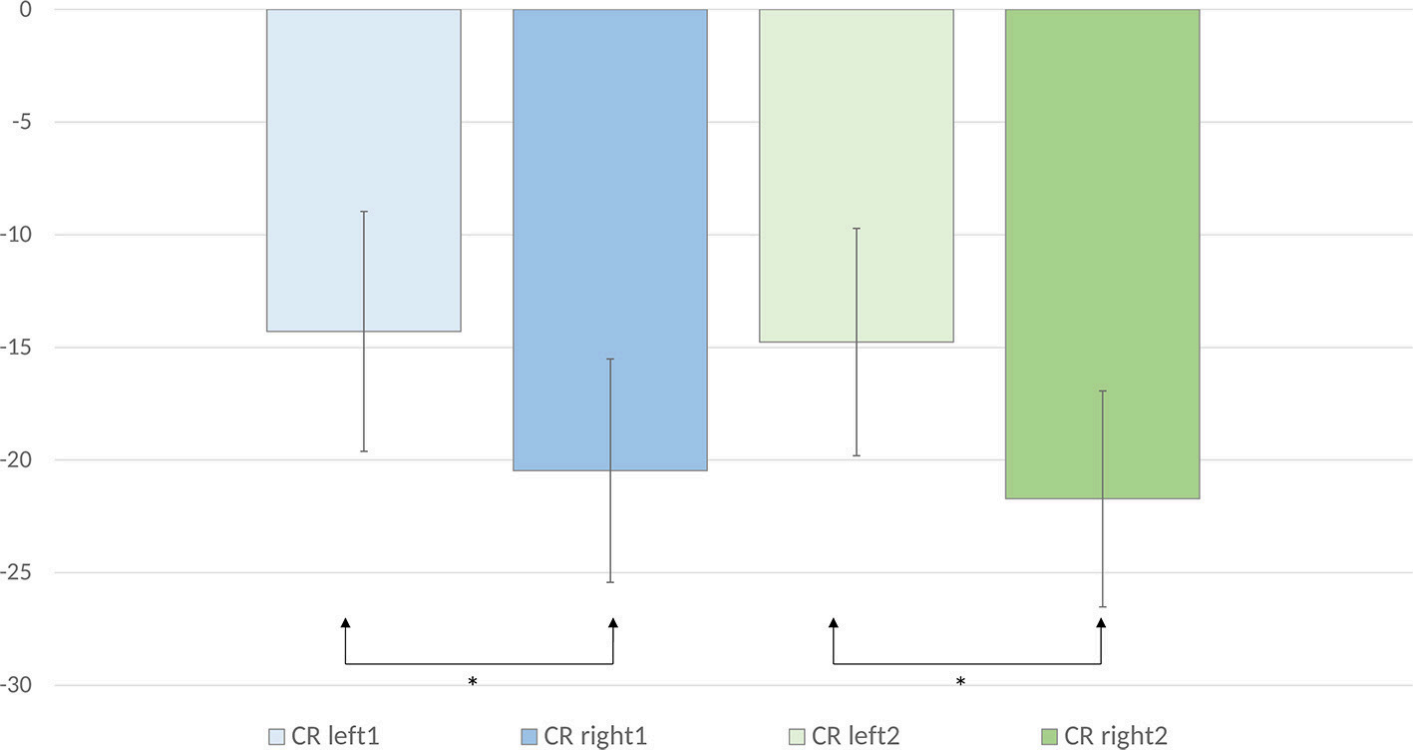
Mean lower alpha ERD values for the creative (alternate use) condition over the left and right frontal and central electrode sites are displayed. Error bars represent standard error values. The mean ERD values for left1 were calculated by averaging the ERD values for F3, FC3, and C3. The mean ERD values for left2 were calculated by averaging the ERD values for F1, FC1, and C1. The mean ERD values for right1 were calculated by averaging the ERD values for F4, FC4, and C4. The mean ERD values for right2 were calculated by averaging the ERD values for F2, FC2, and C2.

An anterior-posterior × laterality × word pair type ANOVA was performed on the ERD values calculated for the evoked and induced power in the lower alpha frequency band. It revealed a significant anterior-posterior × laterality × word pair type interaction [*F*_(40, 840)_ = 2.23, *p* < 0.001, $\eta_{p}^{2}$ = 0.10]. To deconstruct this interaction, we performed separate analyses for each of the anterior-posterior levels. Analyses for centro-parietal, parietal, and parieto-occipital sites did not reveal any significant main effects or interactions [*ps* > 0.05]. Significant laterality × word pair type interactions were observed over frontal [*F*_(8, 168)_ = 3.46, *p* < 0.01, $\eta_{p}^{2}$ = 0.14], fronto-central [*F*_(8, 168)_ = 4.02, *p* < 0.01, $\eta_{p}^{2}$ = 0.16], and central sites [*F*_(8, 168)_ = 2.96, *p* < 0.05, $\eta_{p}^{2}$ = 0.12]. To deconstruct these interactions, we computed mean ERD values across the frontal, fronto-central, and central sites for each laterality level. In this way, we obtained the following levels of the laterality factor: Left1 (mean of F3, FC3, and C3 for each word pair type), Left2 (mean of F1, FC1, and C1 for each word pair type), Mid (mean Fz, FCz, and Cz for each word pair type), Right1 (mean of F4, FC4, and C4 for each word pair type), and Right2 (mean of F2, FC2, and C2 for each word pair type). Mean ERD values for each level and word pair type are displayed in Table 2 and Figure 4. A paired samples *t*-test comparing mean ERD in the lower alpha band calculated for each word pair type over the left and right sites revealed that alternate uses evoked smaller ERD over left than right sites [Left1 (*M* = −14.29, *SE* = 5.32) vs. Right1 (*M*= −20.47, *SE* = 4.96) (*p* = 0.04, *d* = 0.47), Left2 (*M* = −14.76, *SE* = 5.05) vs. Right2 (*M* = −21.72, *SE* = 4.79) (*p* = 0.005, *d* = 0.68)]. The analyses of common uses and unrelated word pairs did not reveal significant differences (*p* > 0.05).

**Table 2:** Mean lower alpha ERD and SE values for common uses, alternate uses, and unrelated word pairs for the following laterality levels: Left1 (mean of F3, FC3, and C3), Left2 (mean of F1, FC1, and C1), Mid (mean Fz, FCz, and Cz), Right1 (mean of F4, FC4, and C4), and Right2 (mean of F2, FC2, and C2).

**Laterality levels**	**Word pair type**	**Mean**	**Standard error**
Left1	common	−16.57	5.41
	alternate	−14.29	5.32
	unrelated	−20.22	3.79
Left2	common	−14.13	4.93
	alternate	−14.76	5.05
	unrelated	−16.56	3.24
Mid	common	−16.43	5.06
	alternate	−17.47	4.92
	unrelated	−14.79	3.60
Right1	common	−19.12	4.61
	alternate	−21.72	4.79
	unrelated	−12.80	4.33
Right2	common	−19.46	4.38
	alternate	−20.47	4.96
	unrelated	−12.24	4.73

Supplementary analyses were performed for the theta and beta frequency bands. No main effects or interactions were observed in the analysis of lower and mid theta power bands (*ps* > 0.05). In the upper theta band, an anterior-posterior × laterality × word pair type ANOVA revealed a significant anterior-posterior × laterality × word pair type interaction [*F*_(40, 840)_ = 1.47, *p* < 0.01, $\eta_{p}^{2}$ = 0.08]. To deconstruct this interaction, we performed separate analyses for each of the anterior-posterior levels. Only the analysis of data recorded over the frontal sites showed a significant laterality × word pair type interaction [*F*_(8, 168)_ = 2.60, *p* = 0.05, $\eta_{p}^{2}$ = 0.11]. The smallest mean ERD values were observed for alternate uses over F1, and a comparison of mean ERD values for each word pair type between F1 and F2 showed that alternate uses evoked smaller ERD values over the F1 (*M* = −9.79, *SE* = 9.87) than F2 (*M* = −20.88, *SE* = 7.33) [*p* < 0.01, *d* = 0.75]. A similar pattern was observed for common uses, with smaller ERD values observed over F1 (*M* = −15.77, *SE* = 8.39) than F2 (*M* = −23.16, *SE* = 7.08) (*p* = 0.01, *d* = 0.58), while no difference was found for unrelated word pairs (*p* > 0.1).

No main effects or interactions were observed in the analysis of lower beta (*ps* > 0.05). An anterior-posterior × laterality × word pair type ANOVA for upper beta band revealed a significant main effect of word pair type [*F*_(2, 42)_ = 5.00, *p* = 0.01, $\eta_{p}^{2}$ = 0.19]. Bonferroni corrected pairwise comparisons showed that common uses evoked the largest ERD in the upper beta band (*M* = −10.52 *SE* = 2.17) and differed from unrelated word pairs which evoked the smallest ERD (*M* = −4.89 *SE* = 2.40) (*p* = 0.04, *d* = 0.58). ERD for alternate uses fell in-between the two word pair types (*M* = −7.85, *SE* = 2.21), but did not significantly differ from them (*p* > 0.05). However, a significant linear effect was observed [*F*_(1, 21)_ = 7.51, *p* = 0.01, $\eta_{p}^{2}$ = 0.26].

## 4. Discussion

In the study reported above, we employed an alternate use evaluation task (AUeT) to investigate whether evaluating alternate uses is related to reduced alpha event-related desynchronization (ERD) and increased N400 amplitudes when compared to evaluating common uses. Such effects would point to increased semantic processing demands and attentional demands related to creative idea evaluation. In the task, participants evaluated word pairs which represented common uses, alternate uses, and unrelated word pairs. The word pair categories were established based on two normative studies performed prior to the EEG experiment.

The main aim of the current study was to test whether alternate use evaluation evokes reduced ERD (more activity) in the alpha band compared to common use evaluation. This effect was found in the upper alpha band over parieto-occipital regions, and was right lateralized. To the best of our knowledge, no study so far has investigated the changes in the upper alpha band in an alternate use evaluation task, for which reason we will interpret them in light of (1) EEG studies on creativity, which employed generation tasks and observed event-related synchronization (ERS) in the alpha frequency band in creative thinking modes, and (2) EEG studies on the relationship between ERD in the upper alpha band and semantic processing.

Previous studies on creative ideation have shown larger ERS in the alpha band on creative than non-creative trials. These studies employed divergent thinking tasks, in which participants generated alternate uses of objects or remote word associations. Also, the time windows used for the EEG analyses spanned over up to several seconds, the period of time over which participants produced their responses. Although the current study is different from most EEG research on creativity in that it employs an evaluation task and examines the time window of up to 1,000 ms after critical word onset, more power in the upper alpha band, here visible as reduced ERD, was also observed. Our results reveal that after an initial release in inhibition following the critical word onset, the observed ERD in the upper alpha band is smaller for creative than for common uses between 400 - 1,000 ms post-stimulus. This effect is observed over parieto-occipital electrode positions. At the same time, alternate uses might be linked to smaller lower alpha ERD over the left than right anterior sites.

Our results are partly in line with findings reported by Benedek et al. (2011), who observed increases in the alpha band (with no differences between upper and lower alpha sub-bands) over right parieto-occipital regions in the divergent as compared to convergent thinking task, under high internal processing demands, and increases in the alpha band over frontal sites linked to high internal processing demands in both divergent and convergent thinking modes. Benedek et al. (2011) interpreted the frontal effect as reflecting general attentional demands, not directly related to creative task demands.

Similar frontal alpha synchronization was reported by Camarda et al. (2018), who observed a positive relationship between frontal alpha and the remoteness of the alternate uses generated by the participants who completed an alternate uses task. Interestingly, the authors split the time windows in the analysis and observed that participants who generated less original uses showed a decrease in frontal alpha synchronization over time, while participants who generated original uses maintained this synchronization over all the time windows analyzed. They interpreted this effect as reflecting more involvement of cognitive control when novel uses are generated and linked it to more efficient inhibition.

In our study, the analysis of data recorded over the frontal sites showed an effect only in the lower alpha ERD. The ERD was smaller over the left than right anterior regions solely for alternate use evaluation, which may be related to top-down inhibition required for the evaluation of alternate uses, but not for common uses or unrelated word pairs. Since the task was the same across the conditions, this effect cannot be explained solely by attentional demands, but seems to be related to evaluating alternate uses.

The latter effect reported by Benedek et al. (2011) was an increase in alpha over the right parieto-occipital sites. They interpreted it as related to recombination of semantically distant information. In a follow-up study, Benedek et al. (2014) noted a similar increase in alpha over the right parieto-occipital areas in an alternate uses task, which was not modulated by internal processing demands. They proposed that because generating alternate uses might involve mental operations related to visual mental imagery and manipulation of generated mental images of objects, such mental operations are demanding and can be sensitive to any interference of task-irrelevant information coming from the visual stream. For this reason, such tasks require task shielding, reflected in the increased activity in the alpha band. The functional role of alpha might be thus to block information coming from the visual stream, so that the mental imagery task can be carried out. A similar increase in the upper alpha band over the right temporo-parietal sites was reported by Schwab et al. (2014), who observed it in the time interval preceding response generation. Interestingly, the more original the generated use, the stronger the asymmetry of the observed effect was. Finally, Camarda et al. (2018) linked alpha synchronization over the temporo-parietal sites (not lateralized) to the novelty of the generated responses, and interpreted it as related to internal semantic processing demands.

In the current study, we observed an effect similar to the one reported by Benedek et al. (2014), however (1) rather than a task difference, we noted a difference between the common and alternate uses, with task demands being equal, and (2) this difference was observed only in the upper, but not lower alpha band, over the right parieto-occipital regions. Although Benedek et al. (2014) used a generation task (alternate use task (AUT)), and we employed an evaluation task (alternate use evaluation task (AUeT)), participants in both tasks possibly imagine either a use they generated (AUT), or a use represented in a word pair, which they need to evaluate (AUeT, employed in the current study). The more demanding mental imagery in the case of alternate uses might be linked to the need to re-represent the object, so that it fits the novel, original use. The mental image of an alternate use is thus more elaborate than a mental image of a common use, requiring the participants to link more distant information and integrate it in the visual image of the novel use. This degree of elaborateness and complexity of re-representation seems to modulate the activity in the upper alpha band over the right parieto-occipital regions in the evaluation task. While the effect observed in the upper alpha band in the current study can be linked to conceptual re-representation and increased semantic processing demands, the effect in the lower alpha band found over anterior sites can be related to increased working memory and attentional demands. Thus, in the alternate use evaluation task, the two sub-bands might be seen as indexing different cognitive operations.

One previous EEG study that used an evaluation condition also revealed increased power in the upper alpha band (Hao et al., 2016). The design consisted of two blocks, in each of which a trial included three stages. In block 1, participants generated alternate uses of a given object, then evaluated them, and then generated and reported the most original idea. In block 2, instead of evaluating the generated ideas, participants performed a distraction task. Increased power in the upper alpha band was found when the evaluation task was compared to a distraction task. Also, of the two idea generation phases, an increase in upper alpha was observed in the second phase, only if it followed the idea evaluation and not the distraction task. These effects were observed over frontal sites and interpreted as reflecting heightened internal attention.

To evaluate whether it is common or uncommon, as well as possible, to use, e.g., a *balloon* as a *packet*, participants need to integrate information related to two remote concepts and discover the features and relations that allow *a baloon* to be used as *a packet*. This recombination of semantic information relates to two notions present in research on semantic processing and creativity, i.e., conceptual expansion and re-representation. Conceptual expansion has previously been discussed in the context of, e.g., novel metaphor comprehension, as a mechanism underlying creating new meaning. When the new meaning is constructed, a concept is expanded. In our case, the concept of, e.g., *a balloon* is now broadened by a new use of a balloon as a packet, and the concept of *a packet* is broadened as a new object, here *a balloon*, can be included in the *packet* category, however low in frequency this use remains. In a similar vein, we can see the mental operations involved in the evaluation of a novel object use as an instance of re-representation. In this case, the representations of *a balloon* and *a packet* change due to the inclusion of a novel use of *a balloon* and a broadened array of objects that can be included in the category of *a packet*. In order to answer a question whether *a balloon* can be used as a *packet*, participants need to discover the similarity between *balloon* and *packet*, an association that has not been formed earlier, and create a re-representation of *a balloon* that will include the function of *a packet* (Batchelder and Alexander, 2012; Olteţeanu and Falomir, 2016). In line with Benedek et al. (2011) and Benedek et al. (2014), more activity in the alpha band might reflect task-shielding necessary for a successful completion of the task. Although not explicitly tested in the current project, creating such a re-representation might pose demands on semantic processing (e.g., feature access and matching) and mental imagery (e.g., object rotation). A potential link between these specific operations and activity in the alpha band needs further investigation with electrophysiological methods.

Several studies outside the scope of creativity have investigated the functional role of the upper and lower alpha sub-bands in the context of semantic memory, episodic memory, and attention (Klimesch et al., 1997a,b; Klimesch, 1999). These studies employed a methodological approach different from paradigms used in research on creativity, with tasks such as semantic congruency judgment or cued-recall. The recorded EEG signal was analyzed within up to 1,000 ms post stimulus. An ERD in the upper alpha band was generally observed in tasks that involved semantic judgments, but not in tasks related to episodic memory. Based on these findings, upper alpha was linked to semantic memory. Our results are in line with these findings, in that we observed an overall ERD in the upper alpha band, but the degree of the ERD differed depending on word pair type. For semantically complex (alternate) uses, the ERD was smaller than for less semantically complex (common) uses.

Our results also partly replicate the results of a semantic priming study (Mellem et al., 2013), which revealed smaller power in the alpha band (8–12 Hz) between 400 and 1,000 ms post-stimulus for semantically unrelated than semantically related word pairs. We have observed a similar difference between common uses and unrelated word pairs. There are, however, several differences that make a direct comparison of the results of the two studies difficult. Mellem et al. (2013) did not use an intermediate category of remotely related items, which would correspond to alternate uses in the current report. Also, they employed a delayed letter-search task, which does not explicitly draw participants' attention to the semantic aspects of the stimuli. Finally, the effect was observed over frontal sites in the alpha band, while our results point to a clear parieto-occipital distribution of the effect in the upper alpha band. Overall, while Mellem et al. (2013) interpreted their results as reflecting attentional processes, the results reported in the current study seem to be more directly linked to semantic processing demands. Specifically, word pairs semantically most difficult to integrate (unrelated word pairs) evoked the smallest ERD, word pairs easiest to integrate (common uses) evoked the largest ERD, and the category representing the intermediate integration difficulty (alternate uses), evoked intermediate ERD in the upper alpha band. Further empirical examination is necessary though to elucidate the functional role of the upper alpha band oscillations in evaluation tasks involving different degrees of semantic relatedness and creativity.

Supplementary analyses in the theta and beta bands, conducted to provide possible reference for both previous and future research on the oscillatory correlates of creativity, revealed effects in the upper theta band and the upper beta band. In the upper theta band, creative and common uses evoked smaller ERD over the left than right anterior sites. Previous studies have shown a relationship between theta and attentional demands, as well as lexical encoding (Klimesch, 1999). More specifically, increases in frontal theta have been linked to working memory demands (Klimesch et al., 1997a). In our results, reduced ERD in the upper theta band might be related to increased working memory load, task demands, or word encoding demands for common and alternate uses. It should be noted, though, that there is a partial overlap in the frequency range between upper theta and lower alpha, and the results in both sub-bands bear some resemblance in that smaller ERD was found over the left than right anterior sites for alternate uses (both lower alpha and upper theta) and for common uses (upper theta only). At the same time, these effects are stronger and more widely distributed (frontal, fronto-central, and central sites) in the lower alpha band than in the upper theta band (frontal sites). Since these effects remain difficult to disentangle, the conclusions should be treated as tentative.

Finally, in the upper beta band a linear trend was found with unrelated word pairs evoking smaller ERD than alternate uses, which in turn evoked smaller ERD than common uses. No topographical differences were found for this effect. Larger beta power has been previously linked to creative task demands in just a few studies. For example, Mölle et al. (1999) found larger beta power on divergent as compared to convergent thinking tasks over central and parietal sites. Danko et al. (2009) also reported larger upper beta power in creative tasks when compared to non-creative tasks. Importantly, the non-creative tasks were controlled for the degrees of complexity, to exclude task complexity as a factor influencing the results. Such findings have, however, not been frequently reported, and further research is needed to account for the relationship between activity in the (upper) beta band and creative thinking demands.

In the analysis of the ERP components, the observed effects confirmed our expectations. Within the late N400 time window (400–500 ms), alternate uses and unrelated word pairs evoked larger N400 amplitudes than common uses. Alternate uses and unrelated word pairs did not differ, but a significant linear effect was observed. Although difference wave maps point to an anterior distribution of the effect, no topographical differences were observed in the statistical analysis. The difference between creative and common uses is in line with the pattern observed by Kröger et al. (2013), but also with the results of several studies on novel metaphor comprehension, in which N400 amplitudes were larger for novel metaphoric (creative) than literal (non-creative) utterances (Arzouan et al., 2007; Lai et al., 2009; Goldstein et al., 2012; Rataj et al., 2018). An increase in the N400 amplitudes was also reported in a recent study on creative advert evaluation, in which the same images presented together with advertising slogans evoked larger N400 amplitudes than those presented with literal descriptions of the images (Zhou et al., 2018). The increase in the N400 amplitudes in response to alternate uses observed in the current study might point to increased demands in semantic information retrieval, related to creating a re-representation or to conceptual expansion. Interestingly, the effects are comparable across different stimulus types (novel metaphors and alternate uses) and different tasks (an alternate use evaluation task vs. a semantic decision task, or a reading task). Although no direct links can be made here due to differences in materials and procedures, this striking similarity points to the importance of unifying the electrophysiological research on the evaluation of various types of creative content, which will in turn help elucidate specific cognitive processes underlying creative thinking.

Between 500 and 1,000 ms after critical word onset, the ERP results showed a sustained negativity effect, with unrelated word pairs evoking the most negative amplitudes, followed by creative and common uses over frontal and fronto-central sites. A significant linear effect was found here. This pattern seems to reflect sustained negativity that started as early as around 400 ms post-stimulus. In the later time window (between 500 and 1,000 ms), however, the amplitudes observed for alternate uses diverged from those recorded for unrelated word pairs. This result is in line with the findings reported by Kröger et al. (2013), and also partly with the results reported by Rutter et al. (2012) in an ERP study that investigated metaphor comprehension with a delayed response task. In this study, participants evaluated whether or not a given metaphoric sentence was unusual (Question 1) and appropriate (Question 2). This sustained negativity effect, with meaningless sentences evoking larger sustained negativity than novel metaphoric and literal sentences, was interpreted as indexing the difficulty in integrating sentence meaning. Although in the two studies no topographical differences were found for the sustained negativity effects, in the current study the effects were clearly anterior, and anterior negativities have previously been linked to increased semantic working memory demands (Steinhauer et al., 2010). In the task we employed, the increased demands on semantic working memory could be triggered by the need to maintain the activated semantic information due to the delayed response procedure, as well as by task complexity. This interpretation is supported by the observation that sustained negativity was present in response to all object use types, and was modulated by the degree of semantic integration difficulty as well as demands related to creating object re-representations, with more difficult items (unrelated word pairs) evoking larger negativity than less difficult ones (common uses), and the amplitudes for novel items (alternate uses) falling in-between.

Finally, behavioral data analyses revealed that common uses evoked longer reaction times than alternate uses and unrelated word pairs. The results were similar when reaction times were calculated for trials selected based on normative data and for those selected on the basis of EEG study participant judgments. This effect is somewhat surprising, as common uses were evaluated as more common and more usable than alternate uses in the normative study, and thus should be easier to evaluate than alternate uses. Two factors might have contributed to this result. First, the common uses created for the current study were selected so that they were more common than alternate uses, but at the same time not common enough to evoke the critical word associations once the first word of a given pair was presented to participants. If participants had been presented with very common uses, e.g., *comb - hairbrush*, the reduced N400 amplitudes for common uses could be explained by expectancy effects, rather than differences in semantic complexity between the common and alternate uses. For this reason, we selected common uses that do not evoke strong associations. Second, the apparent discrepancy between the normative study results and the reaction time results in the EEG experiment might stem from the task itself. While the normative studies were conducted offline, with ample time to complete the task and a single question to answer (either how common, or how usable a given use is), the task in the EEG experiment involved reaction time measurements and responses to two questions. Also, while normative study participants could choose one out of seven options on a scale for each question, the EEG study participants had only two options for Question 1 (*common - uncommon*), and four options for Question 2 (*usable - slightly usable - slightly unusable - unusable*). The time and task complexity factors were therefore different between the normative study and the EEG study, which might have contributed to the effect observed in reaction times.

The comparison of alternate uses and unrelated word pairs showed that reaction times were longer for alternate uses in the analysis based on normative data, and did not differ in the analysis based on EEG participants' responses. However, this difference might stem from the difference in the number of trials, which was considerably lower in the alternate use condition than in the unrelated word pair condition in the analysis based on EEG participants' responses. This difference in the proportion of trials might have masked the effect present in the analysis based on normative study results. Also, accuracy results showed that creative and common uses were evaluated correctly, i.e., in line with the normative data results, less frequently than the unrelated word pairs. Again, these results stem from factors discussed above, and show that both common and alternate uses were more difficult to evaluate than unrelated word pairs. Additionally, previous research on the evaluation of ideas generated by participants in alternate uses tasks has shown that more original ideas are more likely to be expressed with several words rather than a single word (Forthmann et al., 2017). In our study, the context for all the uses is minimal in all cases, for which reason it might be especially difficult to evaluate the common uses, which are not extremely common due to the reasons mentioned above.

The normative study results and stimulus categories selected based on these results were strongly reflected in the observed N400 and sustained negativity patterns. These effects replicate the results reported by Kröger et al. (2013) and show that stimulus selection properly reflected the three stimulus categories. The possibility of using these categories allowed us to avoid bias discussed by VanRullen (2011), and caused by brain activity associated with correct responses rather than stimulus category. Importantly, it also increased the signal to noise ratio in the ERP and time-frequency analyses. In this way, the current approach demonstrates the usefulness of employing normative studies in stimulus selection procedures in EEG research on evaluating creative ideas.

The current study employed an AUeT, an approach that clearly differs from the most widely used AUT in that it does not involve the generation of alternate uses. In our task, participants evaluated ideas that were not their own, which seems to involve searching for information in the semantic network and discovering (or not) the mappings between the two potentially meaningful stimuli presented to the participants. The differences between evaluating one's own as compared to someone else's creative ideas definitely calls for further investigation. However, our main finding regarding alpha band oscillations partly converges with several previous studies that employed generation tasks, which might point to potential similarities between the two apparently different designs, pertaining to the evaluation stage included in several models of creativity. Additional support for this view can be found in a recent study on creativity in the figural domain (Rominger et al., 2018), in which participants generated creative ideas and then elaborated on them, i.e., mentally improved their originality. In this study, idea elaboration, but not generation, was linked to an increase in the upper alpha band over parieto-occipital sites.

In sum, the major finding of the current study is that alternate use evaluation is linked to more activity in the upper alpha band than common use evaluation. Interestingly, this effect had a right-lateralized parieto-occipital distribution, pointing to possible involvement of increased inhibition of information coming from the visual stream that could disturb the processes of object re-representation or recombination of semantic information. The novelty of this finding lies in that it was present in an evaluation task, which emphasizes the need to examine alternate use evaluation as an important aspect of creative ideation. Additionally, the study corroborated previous ERP findings in studies on creative language comprehension, showing increased N400 and sustained negativity amplitudes evoked when participant evaluate creative items, such as alternate uses or novel metaphors. All in all, the current results point to the importance of investigating semantic processing and object re-representation in future electrophysiological research on creativity.

## Author contributions

KR, FvdV, and DN Conception and design of the study, reading, reviewing, and approval of the submitted version. KR Development of methodology, EEG data analysis, programming of the experiment, statistical analysis of the EEG and behavioral data, management and coordination of research activity, writing of the original draft, and visualization. KR and DN Collecting normative data and conducting the experiment. FvdV funding acquisition.

### Conflict of interest statement

The authors declare that the research was conducted in the absence of any commercial or financial relationships that could be construed as a potential conflict of interest.

## Footnotes

^1^Cohen's *d* values reported in the current paper were calculated by $d=\frac{t}{\sqrt{n}}$ (Lakens, 2013).

^2^Since one participant in the current study obtained a mean score of −33 on the handedness test, we performed additional analyses on the upper alpha ERD, in which we excluded data from this participant. All main effects and interactions reported for the complete dataset were also present in these analyses.
